# Diabetic Kidney Disease Represents a Locus of Opportunity

**DOI:** 10.3389/fphys.2021.650503

**Published:** 2021-03-08

**Authors:** Carolyn Mary Ecelbarger

**Affiliations:** Department of Medicine/Division of Endocrinology and Metabolism, Georgetown University, Washington, DC, United States

**Keywords:** diabetes, kidney, diabetic nephropathy, hypertension, renal physiology and pathophysiology

According to the World Health Organization, the global presence of type 2 diabetes (T2D) rose sharply from 4.7 to 8.5% of the population between the years 1980 and 2014 (Emerging Risk Factors Collaboration et al., 2010). Moreover, T2D is associated with a 30–50% risk of diabetic nephropathy (DN) (Gheith et al., 2016). Given a world population of ~7.8 billion (December 2020) (Worldometer, 2020), we may expect as many as 300–400 million affected persons. DN is the leading cause of end-stage renal disease (ESRD) (Toth-Manikowski and Atta, 2015). Currently renal transplantation is the treatment of choice for patients with ESRD due to improvements in graft survival; however, the wait for an available organ may extend to 3–5 years (Allen et al., 2018; Clayton et al., 2018). Dialysis is clearly not the answer, at least as it is currently employed, as the 5-year survival rate for patients receiving some form of hemodialysis hovers only at around 20–40% (Huff, 2020). Moreover, the cost of treating these subjects is phenomenal, and many cannot gain access to or afford any type of treatment. Only a handful of new innovative and efficacious strategies to combat DN has made it to the clinic in the last 50 years. Why is this the case?

It is partly because we do not fully understand the problem. The kidney is clearly a complicated organ derived from three overlapping sequential systems—the pronephros, the mesonephros, and the metanephros, which are all derived from the urogenital ridge (Qiaggin and Kreidberg, 2006). The kidney conducts a variety of seemly unrelated tasks utilizing a variety of specialized cell types extending along the renal tubule. The 3-D architecture of the kidney is essential in its role in regulating whole-body fluid balance, acid-base homeostasis, blood pressure control, excretion of toxic substances, and reabsorption of vital filtered substances. In the process of cleaning the blood, the proximal tubule (PT) is tasked with reabsorbing and recycling a number of substances, e.g., glucose, amino acids, electrolytes, and water, which would otherwise be lost. Urine is concentrated by the use of energy to generate a cortico-medullary sodium and urea gradient in the interstitium, allowing for passive reabsorption of water, regulated tightly by vasopressin. All of these aspects of normal kidney function can be compromised by DN.

DN may be described as a “perfect storm” involving inflammation, fibrosis, and oxidative stress. Histologically, it has a number of features that distinguish it from other forms of renal disease. For example, Kimmelstein-Wilson lesions, composed of nodular, circular, scar tissue, will form in the glomerulus. Other features include an expanded mesangium and increased mesangial cell number in the glomerulus. The basement membranes of the glomerulus and tubules become thicker (up to 3X) with deposition of collagen, albumin, and IgG along their borders. There is an accumulation of the advanced glycation end product (AGES) due to the partially reduced sugar moieties. AGES increases protein cross-linking, inflammation, and oxidative stress. Foot processes in the glomerulus merge. A common theme underlying the pathology of DN can be broadly thought of as dysregulated utilization of energy.

We can treat DN both by treating T2D itself, i.e., tightly controlling blood glucose levels, as well as, via strategies that short-circuit the effects of T2D on the kidney. For purposes of this article, I will focus primarily on the latter. First, I will discuss the specific challenges of DN in regard to mechanisms of the disease, and standards of care. Second, I will discuss some newer medication strategies that are promising. Finally, I will turn to developing approaches to treat the patient when the kidneys have failed, e.g., xenotransplantation, organ regeneration, and portable artificial kidneys.

T2D is associated with a number of physiological changes, the most obvious being hyperglycemia. One of the main targets of hyperglycemia in the kidney, as well as other organs, is the vasculature (Magee et al., 2017). Endothelial dysfunction, impaired nitric oxide generating capacity, as well as, atherosclerotic plaque formation contribute to impaired blood flow and altered renal hemodynamics. Hyperglycemia is also associated with hyperinsulinemia early in the course of T2D (Tiwari et al., 2007b). We have shown insulin receptor expression is reduced in the cortex and medulla of the kidney in hyperinsulinemic, obese, and T2D rats (Tiwari et al., 2007a). Nonetheless, some aspects of insulin signaling may remain intact in the kidney, in particular those acting through the insulin receptor substrate type 2 (IRS-2) (Ecelbarger, 2020). This can lead to inappropriate upregulation of sodium reabsorption and gluconeogenesis in the PT (Nakamura et al., 2015, 2019).

In the kidney *per se*, the metabolism of epithelial and endothelial cells is altered in an environment in which glucose levels are higher than the norm, i.e., 5.5 mM. Elevated cellular glucose levels lead to a rise in oxidative phosphorylation by the mitochondria. This liberates oxidative radicals including superoxide, which may overwhelm the normal anti-oxidative complement of the mitochondria, e.g., manganese superoxide dismutase (Burgos-Moron et al., 2019). Reactive oxygen species (ROS) can damage cellular DNA, lipids, and proteins, as well as other organelles, such as the endoplasmic reticulum (ER). It appears that mitochondrial DNA, which codes for many of the components of the electron transport chain, is particularly susceptible to ROS damage (Burgos-Moron et al., 2019). Damage to the ER can lead to misfolded proteins, a critical step in their biogenesis (Zeeshan et al., 2016).

Inflammation in DN is initiated as a protective response to early tissue injury or cell death (Perez-Morales et al., 2019). Major players in the inflammation associated with DN (which can include both systemic and localized aspects) include activation of macrophages, the nuclear transcription factor (NFκb), the Janus kinase/signal transducers and activators of transcription (JAK/STAT) pathway, and inflammatory cytokines. Inflammatory cytokines include interleukins-1, -6, 18, and tumor necrosis factor, alpha (TNF-α). Chemokines, which attract other inflammatory molecules as part of their actions, include monocyte chemotactic protein-1 (MCP-1) and a number of chemokine C-C motif ligands, e.g., CCL2, CCL5, and CX3CL1. There are currently a number of anti-inflammatory approaches to treat DN in various stages of clinical trials (Perez-Morales et al., 2019). For example, pentoxifylline (PFA) targeting cytokines (Navarro-Gonzalez et al., 2018), baricitinib, a JAK 1/2 inhibitor (Tuttle et al., 2018), CCX140-B, a CCR2 inhibitor (de Zeeuw et al., 2015), and emapticap pegol, an MCP-1/CC2 antagonist, have all been shown to reduce albuminuria in separate clinical studies (Menne et al., 2017).

Hypertension is a major risk factor for the development of DN (Lovshin and Cherney, 2014). Hyperglycemia likely affects blood pressure through a number of indirect means. Increased pressure at the level of the glomerulus causes podocyte effacement and, eventually, nephron dropout, which consequently increases the load on remaining nephrons. In line with this, inhibitors of the renin-angiotensin-system (RAS) are the current medical “therapy of choice” for the treatment of DN. Several clinical trials have demonstrated a protective effect to reduce albuminuria in hypertensive/proteinuric patients as the result of treatment with angiotensin-converting-enzyme inhibitors, e.g., captopril or angiotensin receptor blockers (ARBs), e.g., losartan (Brenner et al., 2001; Amann et al., 2003). Dual treatment with both classes of drugs did not seem to provide any additive effect (Imai et al., 2013). Other more recent drug combinations, such as aliskiren (renin blocker) with ACEs or ARBs, did not show added benefit and may even have been harmful (Cully, 2013).

Hyperfiltration, supraphysiologically elevated glomerular filtration rate, i.e., GFR >135 ml/min/1.73m^3^, is found to occur early in the course of T2D (Tonneijck et al., 2017), and advances DN. Hyperfiltration may be due to an influx of amino acids in the renal circulation (especially those that promote gluconeogenesis) (Tuttle and Bruton, 1992) or the result of altered tubuloglomerular feedback (TGF), i.e., increased glucose reabsorption in the PT leading to increased sodium reabsorption at this site (Vallon and Komers, 2011). The net result is less sodium at the macula densa, which releases neurohumoral factors via the juxtaglomerular apparatus (JGA), resulting in changes in the pre- or post-glomerular arteriolar tone (Tonneijck et al., 2017). This increases GFR and allows for some sodium to travel to the distal tubule. Other factors present in T2D that may alter the glomerular arteriolar tone include insulin, cyclooxygenase 2 (COX2), angiotensin II, and atrial natriuretic peptide. Despite an abundance of evidence that hyperglycemia is upstream of a plethora of pathways that have been shown to be damaging to the kidney, there is little evidence to show that strict glucose control alone, in the clinical setting, can reverse pre-existing DN (Wong et al., 2016).

SGLT2 (sodium glucose transporter, type 2) inhibitors represent a surprising and major breakthrough in the treatment of DN (DeFronzo et al., 2017). The SGLT2 protein is expressed in the brush border of the S1–S2 portions of the proximal tubule, and considered to be a high-capacity, low-affinity transporter (Poulsen et al., 2015). It appears, somewhat in series, with the SGLT1 isoform, in the S2–S3 segments, and not a target for the inhibitors. The original aim of this drug class, as developed, was to design a safer drug with similar properties to Phlorizin (blocked both isoforms, but which had intestinal side effects) (Rieg and Vallon, 2018). The goal was to deplete circulating glucose levels in the blood by blocking its reabsorption in the kidney; however, the drugs have subsequently been demonstrated to have protective actions independent of their ability to reduce blood glucose, effects that are not clearly understood. A recent meta-analysis to assess cardiovascular and kidney outcome of all 4 available SGLT2 inhibitions, i.e., canagliflozin, empagliflozin, dapagliflozin, and ertuglifozin, in T2D patients demonstrated a consistent improvement in kidney composite outcomes (Hazard ratio = 0.62) across the class (McGuire et al., 2021).

One challenge to overcome is that it is difficult to diagnose DN in its most nascent stages. The presence of microalbuminuria (30–300 mg albumin excreted/day) is an early indicator not only of the presence of renal compromise but also correlates to increased cardiovascular risk (Xia et al., 2015; Al-Rubeaan et al., 2017). Unfortunately, the presence of microalbuminuria is less predictive of the progression of DN in T2D, as compared to T1D (Molitch et al., 2004). There is a need to develop and validate more sensitive biomarkers. The presence of certain microRNAs in urine exosomes may be promising to detect early cellular stress and alterations in fibrotic and autophagic pathways (Ma et al., 2019). Other promising protein-based biomarkers include serum cystatin C (to estimate GFR), urinary angiotensinogen and ACE2, plasma copeptin, serum amyloid A, and serum and urinary zinc α2 glycoprotein (ZAG) (Colhoun and Marcovecchio, 2018).

Other new areas of research include finding and testing direct targets of hyperfiltration, inflammation, and fibrosis (Alicic et al., 2017). A protein kinase C (PKC) inhibitor, ruboxistaurin, was found to reduce albuminuria in T2D (Tuttle et al., 2005). Niclosamine, developed originally as an antihelmintic (tapeworm medicine), is currently in the recruiting stage for a clinical trial for DN (Chen et al., 2018; Mook et al., 2019). Underlying its mechanism of action includes uncoupling of oxidative phosphorylation, and modulation of the Wnt/β-catenin, mTORC1, STAT3, NF-κB, and Notch signaling pathways (Chen et al., 2018). Other new drugs being tested in clinical trials include magnesium supplementation and LMB763, a farnesoid X receptor agonist (U.S. National Library of Medicine, 2020).

There are also technological advances on the horizon for those needing renal replacement therapy (RRT) or those with ESRD (Dang et al., 2020). One area of research is in non-cell-based kidney replacement primarily in the form of wearable artificial kidneys (WAK) and implantable artificial kidneys (IAK) (Salani et al., 2018). Technologically, there were a number of barriers to overcome in making these devices preferable to conventional dialysis at a center, e.g., the volume of fluid needed, power requirements, and weight. Wearable devices in clinical trials currently are <5 kg, and this is accomplished by recycling the fluid using a column that contains urease to hydrolyze urea into ammonia and carbon dioxide (Salani et al., 2018). IAK have not yet advanced beyond animal model systems. The current system in development uses cardiovascular pressure and chemical energy of cellular metabolism to derive power. Also, no dialysate is required as patients consume an electrolyte-rich fluid. Limitations include the invasive procedure to implant the device, and the lifespan of each device is uncertain.

Another possibility gaining traction is to develop a biological kidney prototype, or a tissue-regenerated kidney (Dang et al., 2020). Bioengineered kidneys are generally designed by applying cells to a biologic or partially synthetic scaffold so that the cells, hopefully, given the right spatial cues, reseed and grow a filtering, homeostatic, device similar to the native kidney. Success at this level would be wonderful and assuage the global demand for RRT; however, this may be a ways off, as the kidney is a highly complex organ with more than 26 specialized cell types (Nishinakamura, 2008). A perhaps more realistic short-term approach involves xenotransplantation of “humanized” organs from transgenic animals (e.g., pigs; Peired et al., 2020); however, ethical issues surround this approach.

In conclusion, DN represents a complex disease. It is not only difficult to detect in the early stages, but it is difficult to slow once it is initiated. While dietary management (low Na+, K+ diets) and ACEs and ARBs have been demonstrated to slow the progression, the staggering numbers of currently affected individuals worldwide, as well as those not able to access even these basic therapies, indeed make it a Grand Challenge in the kidney physiology field. We need to dream big and challenge ourselves to find novel approaches to address this often silent killer.

## Author's Note

Grand Challenge, authored by the Specialty Chief Editor of the Renal and Epithelial Physiology section.

## Author Contributions

The author confirms being the sole contributor of this work and has approved it for publication.

## Conflict of Interest

The author declares that the research was conducted in the absence of any commercial or financial relationships that could be construed as a potential conflict of interest.
